# Oral health knowledge, attitude, and practices of adolescents living with HIV in Ibadan, Nigeria: a cross-sectional study

**DOI:** 10.1186/s12903-026-09568-w

**Published:** 2026-08-11

**Authors:** Adeola Temitope Williams, Folake Barakat Lawal, Babatunde Oluwatosin Ogunbosi, Akinyele Olumuyiwa Adisa, Joel Oshogeriamhe Williams, Obafunke Olufunlayo Denloye

**Affiliations:** 1 Department of Child Oral Health, Faculty of Dentistry, University of Ibadan and University College Hospital, Ibadan, Oyo State Nigeria; 2 Consortium for Advanced Research Training in Africa (CARTA), APHRC, Nairobi, Kenya; 3 Department of Periodontology and Community Dentistry, University of Ibadan and University College Hospital, Ibadan, Oyo State Nigeria; 4 Department of Paediatrics, Faculty of Clinical Sciences, University of Ibadan and University College Hospital, Ibadan, Oyo State Nigeria; 5 Department of Oral Pathology, Faculty of Dentistry, University of Ibadan and University College Hospital, Ibadan, Oyo State Nigeria; 6 Department of Child Oral Health, University College Hospital, Ibadan, Oyo State Nigeria

**Keywords:** Oral health awareness, Perception, Oral health behaviour, Socio-demography, HIV, Young people, Infectious diseases

## Abstract

**Background:**

Dental caries, gingivitis, and periodontitis are prevalent in Adolescents Living with HIV (ALHIV). These conditions are highly preventable through the adoption of healthy oral health practices. However, the oral health knowledge, attitude, and practices of ALHIV are underexplored. This study therefore aims to assess the Oral Health Knowledge, Attitude and Practices (OHKAP) of ALHIV in Ibadan, Nigeria and their associated sociodemographic factors.

**Methods:**

This was a cross-sectional study conducted among ALHIVs aged 10–15 years who were receiving care at two selected paediatric infectious disease clinics in Ibadan, Nigeria. Data on participants’ socio-demographic characteristics and OHKAP were collected using a pretested and validated self-administered questionnaire. Mean (± SD) Oral Health Knowledge (OHK), Oral Health Attitude (OHA), and Oral Health Practices (OHP) scores were calculated, and proportions of those with adequate/inadequate knowledge, positive /negative attitude, and good/ poor oral health practices were reported. Binary logistic regression was done to determine the factors associated with OHK, OHA, and OHP.

**Result:**

Data from 195 participants with a mean age of 12.9 ± 1.6years was included in this analysis. There were more males (105, 53.8%) than females. All the adolescents except one (0.5%) were students, and more than half (116, 59.5%) had received prior oral health education, with school being the most cited source of information. The median OHK score (Q_1_, Q_3_) was 22.2(11.1, 33.3) while mean (± SD) OHA and OHP scores were 71.7 (± 0.7) and 44.7(± 9.5) respectively.

While those who were younger and those whose parents had no tertiary education had lower odds of having adequate OHK and good OHP, age was the only factor significantly associated with OHA after adjustment for other variables (AOR = 2.19, 95% CI 1.08–4.41, *p* = 0.03).

**Conclusion:**

This study found that although more than half of ALHIV in Ibadan, Nigeria, had participated in school-based oral health education, the majority of them had inadequate oral health knowledge and engaged in poor oral health practices. However, most of them had a good attitude towards oral health. Age was the only sociodemographic factor significantly associated with attitude to oral health, with younger children having better oral health attitude.

## Introduction

In Nigeria, about 120 000 Adolescents are living with HIV(ALHIV), and this constitutes the largest in West and Central Africa [1, 2]. The presence of HIV infection has been associated with an increased prevalence of oral diseases such as oral mucosal lesions, dental caries, gingival and periodontal disease [3–6].

Documented prevalences of these conditions in ALHIV vary globally [5]. In Kenya, at least about 83% of CALHIV had at least one oral disease, which is similar to findings from China [4, 7]. In Nigeria, about 65% of children and adolescents living with HIV have been reported to have oral diseases [8, 9]. Although with the commencement of the Highly Active Antiretroviral Therapy (HAART), a reduction in the prevalence of oral mucosal lesions has been reported, dental caries and gingival diseases are still common among them [3, 10].

Dental caries, gingival, and periodontal diseases are, however, highly preventable. The adoption of appropriate oral health practices, such as brushing twice daily with fluoridated toothpaste, using appropriate cleaning aids, adopting correct brushing technique, and reducing consumption of cariogenic foods, can help reduce plaque accumulation, a major risk factor for these diseases [11, 12]. Additional risk factors for these conditions, such as a compromised immune system, the use of antiretroviral syrup, and its associated xerostomia, have been postulated for ALHIV. Lam et al. (2022), in their systematic review, posited that the presence of the HIV infection in itself may not be a predisposing factor. Rather, inadequate oral hygiene practices and frequent consumption of cariogenic diets may play a critical role [5].

Meanwhile, the oral health practices of ALHIV are underexplored, with the few available reports documenting poor oral health practices amongst them [7, 9]. Worse still, there is no literature assessing their oral health knowledge and attitude despite the association between these and oral health practices, thus making this study important.

Furthermore, adolescence is a critical transitional period between childhood and adulthood, during which lifelong health behaviours are established [13]. This stage is particularly significant for adolescents living with HIV (ALHIV), who must manage a chronic systemic condition throughout their lives. The added burden of HIV infection, alongside the developmental and psychosocial changes typical of adolescence, increases their vulnerability to poor health outcomes, including oral diseases [13, 14]. Consequently, this period presents a strategic opportunity to adopt and reinforce preventive oral health behaviours. Targeted oral health promotion at this stage may therefore help establish sustainable oral health habits and improve long-term oral health outcomes among ALHIV [15]. 

Therefore, this study assessed the oral health knowledge, attitude, and practices of adolescents living with HIV in Ibadan, Nigeria, and their associated factors. Its findings will aid in developing appropriate oral health educational interventions specifically tailored to improve oral health outcomes in these adolescents.

## Methods

### Study setting

This study was conducted at two paediatric infectious disease clinics (one federal and one state-owned) in Ibadan metropolis, Southwestern Nigeria. Ibadan, the capital of Oyo State, is the largest city in West Africa, spanning 3080 square kilometres. These two centres were selected because they were the main clinics in the metropolis offering paediatric HIV care at the time the study was carried out, and they both serve as referral health care centres to people of diverse socioeconomic strata within the metropolis.

### Study design and period

A cross-sectional study design was adopted, and it was carried out between February and April 2025. A consecutive sampling technique was used, and a total of 195 adolescents aged 10–15 years living with HIV infection and receiving care at these centres were recruited.

**Fig. 1:**
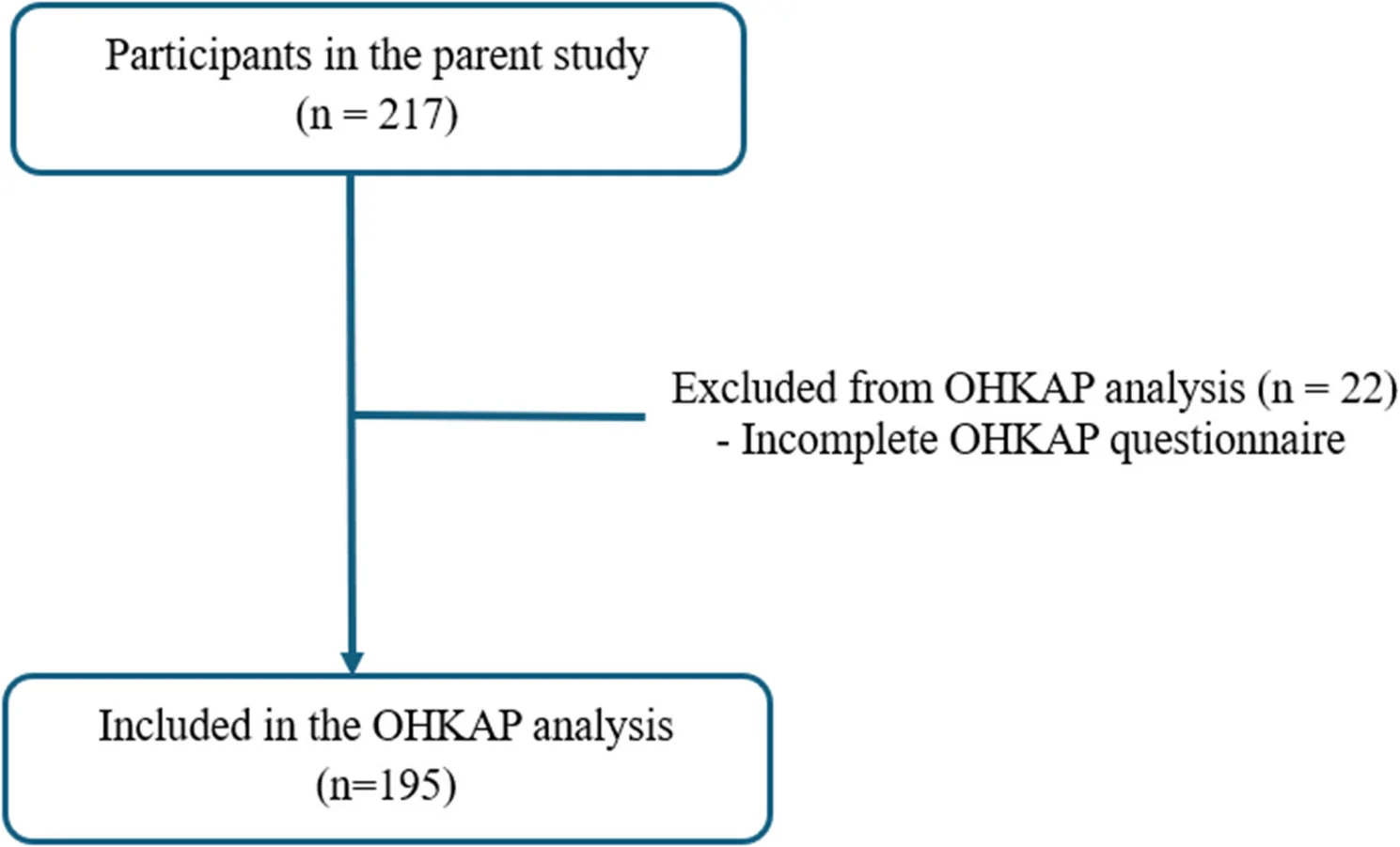
Participants’ flow chart

A minimum sample size was obtained using the formula for sample size estimation for descriptive studies [16]. and adopting a proportion of 8.3%, not involved in any form of oral hygiene cleaning, reported in a previous study [9], a 5% acceptable error margin and a 95% confidence interval. This prevalence was chosen as a proxy for the prevalence of poor oral health practices due to scarcity of literature on the OHKAP of ALHIV, which were the major outcomes for the study. The calculated sample size was further adjusted for a 20% non-response rate, resulting in a minimum required sample size of 147 participants. However, to maximize resource efficiency and improve statistical power, all 195 adolescents with a complete OHKAP data among the 217 recruited for the Dental Care Matters in Children and Adolescents Living with HIV (DECAM-CALHIV) project were included in this analysis. Figure 1. Only the adolescents who were willing to participate and whose parents provided consent were included in the study; those who were acutely ill or had any systemic illness were excluded.

### Data collection

Data were collected as part of the DECAM-CALHIV project conducted among children and adolescents living with HIV and their caregivers in Ibadan, Nigeria. Ethical approval for the study was obtained from the University of Ibadan/University College Hospital review committee (UI/EC/24/0789) and the Oyo State Ministry of Health Research Ethics Committee (NHREC/OYOSHRIEC/10/11/22). Written informed consent and assent were obtained from the accompanying parent or guardian and each adolescent, respectively. Data were collected using a self-administered questionnaire, which had been previously validated in the same environment [17]. The questionnaire was pretested among 20 adolescents at the same hospital, who were not part of the main study. It was divided into four sections: Section A - Sociodemographic characteristics; Section B - Oral health knowledge; Section C - Oral health practices, and Section D - Attitude to Oral health.

Sociodemographic data obtained include age, gender, occupation, class, level of education, parents’ occupation, living conditions, and birth rank. The socioeconomic status of each adolescent was derived using an index used in previous studies in the same environment [18–20]. This index uses multiple items: the father’s occupation, level of education, and the mother’s level of education. In cases where the child lived with a single parent, data from the available parent were used. Where both parents were deceased, data from the child’s legal guardian were used. In addition, the occupation of the parents was classified into skilled and unskilled, as reported in a previous study [17]. The higher occupational class of the two parents/guardians, or that of the only parent available, was recorded for each participant.

Oral Health Knowledge (OHK) was assessed using a mixture of nine (9) open and closed-ended questions, including the number of deciduous and permanent teeth, causes of tooth decay, gum diseases, and the function of fluoride. Oral Health Attitude (OHA) was assessed using fifteen (15) questions that pertained to oral hygiene practices, tooth decay, gingival diseases, dental visits, and consumption of sugary food substances. Lastly, Oral Health Practices (OHP) were assessed using twelve (12) questions on tooth cleaning aids, and frequency of tooth cleaning, including flossing; frequency of consumption of sugar-containing food daily, and pattern of dental visits.

### Study measures

For the assessment of OHK, a score of 1 was given for each correct response, while incorrect and ‘I don’t know’ responses were scored zero (0). The minimum and maximum obtainable scores for OHK were 0 and 9, respectively. In assessing OHA, each item was rated on a five-point Likert-type scale ranging from “strongly disagree” to “strongly agree”, with the intermediate response options of “disagree”, “undecided”, and “agree”. A score of 1 was assigned to the most negative response, and a score of 5 to the most positive response. The minimum and maximum obtainable OHA scores were 15 and 75, respectively. Similar to the assessment of OHK, a score of 1 was assigned to each healthy practice and zero (0) for unhealthy habits, with an obtainable score range of 0–12. Subsequently, the OHK, OHA, and OHP scores were converted to percentages for ease of comparison with other studies.

### Data analysis

The data collected were analysed using IBM Statistical Product and Service Solutions (SPSS) software version 27. Normality of the data was assessed using the Shapiro–Wilk test, visual inspection of Q–Q plots, and evaluation of skewness and kurtosis; the results indicated that the data were normally distributed. Means and standard deviations were used to summarize continuous variables such as age, OHK, OHA, and OKP percentage scores. For further analysis, participants’ age, OHA and OHP scores were categorised around the mean. Age into ‘10-12yrs’ and ‘13–15 years’; OHA percentage score ≤ 71.7 as “negative attitude” and > 71.7 as “positive attitude”, and OHP percentage score ≤ 44.7 as “poor practice” and > 44.7 as “good practice”. For oral health knowledge, those with percentage OHK scores below or equal to 50% were considered to have “inadequate knowledge,” while those with scores higher than 50% had “adequate knowledge”. Positive attitude’ was therefore defined as an OHA percentage score of > 71.7, while ‘Negative attitude’ was defined as an OHA percentage score ≤ 71.7. ‘Good practice’ was defined as an OHP percentage score of > 44.7, while ‘poor practice’ was defined as an OHP percentage score of ≤ 44.7. For oral health knowledge, ‘adequate knowledge’ was defined as an OHK percentage score of > 50, while ‘inadequate knowledge’ was defined as percentage OHK scores below or equal to 50. Frequencies of categorical variables such as gender, socioeconomic status, parents’ level of education and occupation; adolescents’ level of knowledge, attitude, and practices were also reported. Variables with a p-value less than 0.20 in the bivariate analysis and those previously reported to be associated with OHK, OKA, and OHP were entered into multivariate analysis to assess their impact on the dependent variable. This threshold was adopted to avoid excluding potentially important confounding variables from the final model [21]. Multivariable logistic regression analysis was performed to identify factors independently associated with oral health knowledge (OHK), oral health attitude (OHA), and oral health practices (OHP). Statistical significance was set at *p* < 0.05. However, because only a small proportion of participants had adequate oral health knowledge (OHK), Firth’s penalized logistic regression was used to identify factors associated with adequate OHK. This was chosen because it uses a penalized likelihood approach that reduces small-sample bias and provides more reliable coefficient estimates and confidence intervals. The goodness-of-fit of the binary logistic regression models was assessed using the Hosmer–Lemeshow test. The test yielded p-values of 0.36 and 0.95 for the oral health attitude and oral health practice models, respectively, indicating adequate model fit for both models. In addition, multicollinearity among the explanatory variables was assessed using the Variance Inflation Factor (VIF). All VIFs were below 5, with a mean VIF score of 3.13, which was below the commonly accepted threshold and indicated no evidence of substantial multicollinearity among the predictor variables. The crude and adjusted odds ratios (CORs and AORs) with the 95% confidence intervals (CIs) were reported to quantify the strength of associations.

## Result

A total of 195 participants aged 10 to 15years with complete OHKAP responses were included in this analysis; the mean age was 12.9 ± 1.6years. There were more males (105, 53.8%) than females. All adolescents (194, 99.5%) were students, except for one, who was an apprentice. Out of those in school, the majority (147, 75.7%) were in secondary schools. About 40% of the participants belonged to the lower socioeconomic status.

**Fig. 2:**
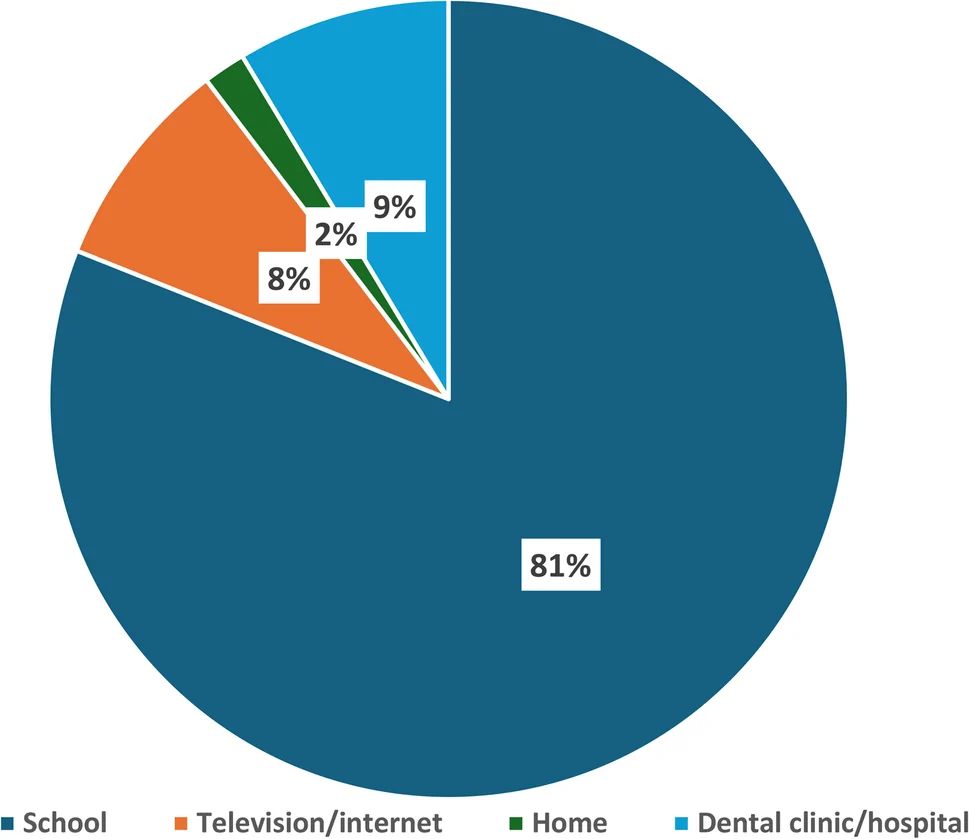
Source of previous oral health information

The majority (184, 94.4%) of the participants had vertical transmission of the virus, (9, 4.6%) had horizontal transmission, while the mode of transmission of two participants was unknown. All of them had already commenced on HAART, with the mean duration of commencement being 93.3 ± 3.5 months. About 60% of them had received previous oral health education, out of which 94 (81%) got the information from schools. Table 1; Fig. 2.

**Table 1:** Socio-demographic characteristics of participants

Characteristic	*n* (%)
Gender	
Male	105 (53.8)
Female	90 (46.2)
Age (years)	
10–12	75 (38.5)
13–15	120 (61.5)
Birth rank	
First child	33 (16.9)
Last child	62 (31.8)
Only child	23 (11.8)
Others	77 (39.5)
Occupation	
Student	194 (99.5)
Apprentice	1 (0.5)
Current school*	
Primary school	47 (24.1)
Junior Secondary	95 (48.7)
Senior Secondary	52 (26.7)
Living condition	
Lives with one or both parents	158 (81.0)
Lives with guardian (orphaned)	19 (9.7)
Lives with guardian (one/both parents alive)	18 (9.3)
Socio-economic status	
Upper	60 (30.8)
Middle	59 (30.3)
Lower	76 (39.0)
Parental level of education	
Less than tertiary	131 (67.2)
Tertiary	64 (32.8)
Highest parental occupation	
Unskilled	97 (49.7)
Skilled	98 (50.3)
Prior oral health information	
Yes	116 (59.5)
No	79 (40.5)

*n = 194 (one participant is not in school)

Concerning their OHK, the percentage score ranged from 0 to 77.8% with a median (Q_1_, Q_3_) of 22.2(11.1, 33.3) and a mean (±SD) of 19.83 (±15.28). Only 8 (4.1%) had an OHK score of 50% and above. The majority 163(83.6%) did not know any cause of gingival/ periodontal disease, while 59(30.3%) could not mention at least one correct cause of dental caries. Only 30(15.4%) had heard about fluoride, but only one participant knew the correct function of fluoride.

The percentage OHA score ranged between 25.0 and 97.0%, with a mean (± SD) of 71.7 (± 0.7), and about 60% of the participants had a positive attitude towards oral health.

The mean (± SD) percentage OHP score was 44.7 (± 9.5), with scores ranging from 16.7% to 83.3%. All the children used toothpaste and a toothbrush, and only one participant used both a toothbrush and a chewing stick. Only 55 (28.2%) brushed their teeth twice daily, of whom 4 participants brushed after breakfast and dinner, and 41 (21%) used a medium-textured toothbrush. Ninety-eight (50.3%) changed their toothbrush every three months, and three participants shared their toothbrush with their siblings. As regards diet, 81 (41.5%) took sugar-containing food substances twice or more daily.

In the bivariate analysis (*p* < 0.2), parental level of education and socioeconomic status were the only factors significantly associated with all three outcome variables (Tables 2, 3 and 4). Participants whose parents did not have tertiary education had lower odds of having adequate oral health knowledge (COR = 0.29; 95%CI 0.07–1.16; *p* = 0.08), positive attitude towards oral health (COR = 0.61; 95%CI 0.32–1.15; *p* = 0.12) and good oral health practices (COR = 0.28; 95CI 0.15–0.53; *p* < 0.001). Likewise, participants who belonged to the upper socio-economic class were about four times more likely to have good oral health practices. (COR = 3.68; 95%CI 1.80–7.50; *p* < 0.001). Tables 2, 3 and 4.

**Table 2:** Firth penalized logistic regression analysis of association between participants’ characteristics and oral health knowledge

	Variable	Oral health knowledge			
		Crude OR	95% CI (*p* value)	Adjusted OR	95% CI (*p* value)
Age	10–12	0.30	0.05–1.80 (0.190)*	0.41	0.06–2.47 (0.332)
	13–15	1.00		1.00	
Gender	Male	2.31	0.52–10.22 (0.269)	2.53	0.59–12.41 (0.199)
	Female	1.00		1.00	
School	Primary	0.16	0.01–2.98 (0.225)	0.26	0.01–5.09 (0.381)
	Secondary	1.00		1.00	
Living condition	Lives with guardian	1.65	0.36–7.43 (0.513)	2.10	0.4–11.1 (0.378)
	Lives with one or both parents	1.00		1.00	
Socioeconomic status	Upper	2.95	0.63–13.70 (0.167)*	2.29	0.07–68.56 (0.632)
	Middle	0.76	0.09–5.95 (0.797)	0.91	0.10–7.98 (0.933)
	Lower	1.00		1.00	
Parental level of education	Less than Tertiary	0.29	0.07–1.16 (0.082)*	0.79	0.04–14.77 (0.879)
	Tertiary	1.00		1.00	
Parental occupation	Unskilled	0.62	0.15–2.47 (0.508)	0.82	0.17–3.93 (0.810)
	Skilled	1.00		1.00	
Previous oral health information	No	0.54	0.12–2.42 (0.429)	0.56	0.12–2.60 (0.468)
	Yes	1.00		1.00	

*OR* Odds ratio, *CI* Confidence interval

*statistically significant at *p*< 0.2

**Table 3:** Logistic regression analysis of association between participants’ characteristics and oral health attitude

	Variable	Attitude towards oral health			
		Crude OR	95% CI (*p* value)	Adjusted OR	95% CI (*p* value)
Age	10–12	2.41	1.29–4.50 (0.006) *	2.19	1.08–4.41 (0.03) **
	13–15	1.00		1.00	
Gender	Male	0.911	0.51–1.6 (0.75)	0.89	0.43–1.46 (0.46)
	Female	1.00		1.00	
School	Primary	1.56	0.78–3.12 (0.21)	1.2	0.55–2.72 (0.63)
	Secondary	1.00	1.00	1.00	
Living condition	Lives with guardian	0.8	0.39–1.67 (0.56)	1.10	0.49–2.46 (0.81)
	Lives with one or both parents	1.00		1.00	
Socioeconomic status	Upper	2.05	1.01–4.15 (0.047)**	1.64	0.31–8.86 (0.57)
	Middle	1.85	0.92–3.7 (0.09)*	1.87	0.86–4.04 (0.11)
	Lower	1.00		1.00	
Parental level of education	Less than Tertiary	0.61	0.32–1.15 (0.12)*	0.66	0.14–3.04 (0.59)
	Tertiary	1.00		1.00	
Parental Occupation	Unskilled	0.90	0.51–1.60 (0.73)	1.15	0.55–2.72 (0.68)
	Skilled	1.00		1.00	
Previous oral health information	No	1.27	0.71–2.3 (0.4)	1.53	0.81–2.91 (0.19)
	Yes	1.00		1.00	

*OR* Odds ratio, *CI* Confidence interval

*statistically significant at *p*< 0.2

** statistically significant at *p*<0.05

**Table 4:** Logistic regression analysis of association between participants’ characteristics and oral health practice

	Variable		Oral health practice		
		Crude OR	95%CI (*p* value)	Adjusted OR	95% CI (*p* value)
Age	10–12	0.81	0.45–1.46 (0.48)	0.87	0.44–1.74 (0.70)
	13–15	1.00		1.00	
Gender	Male	1.04	0.59–1.8 (0.89)	1.05	0.57–1.94 (0.88)
	Female	1.00		1.00	
School	Primary	0.52	0.26–1.05 (0.068)*	0.69	0.31–1.55 (0.37)
	Secondary	1.00		1.00	
Living condition	Lives with guardian	0.87	0.42–1.8 (0.71)	1.12	0.49–2.50 (0.81)
	Lives with one or both parents	1.00		1.00	
Socioeconomic status	Upper	3.68	1.80–7.50 (< 0.001)*	0.97	0.18–5.19 (0.97)
	Middle	1.36	0.66–2.8 (0.41)	1.19	0.54–2.61 (0.67)
	Lower	1.00		1.00	
Parental level of education	Less than Tertiary	0.28	0.15–0.53 (< 0.001)*	0.30	0.07–1.3 (0.13)
	Tertiary	1.00		1.00	
Parental occupation	Unskilled	0.58	0.33–1.04 (0.07)*	0.77	0.4–1.5 (0.44)
	Skilled	1.00		1.00	
Previous oral health information	No	0.70	0.39–1.26 (0.24)	0.82	0.43–1.57 (0.56)
	Yes	1.00		1.00	

*OR* Odds ratio, *CI* Confidence interval

*statistically significant at *p*< 0.2

However, after adjusting for the other variables, only age remained significantly associated with the oral health attitude. The younger children were twice as likely to have a better attitude to oral health than the older age group (AOR = 2.19; 95% CI = 1.08–4.41; *p* = 0.03). Tables 2, 3 and 4.

## Discussion

This study found that the majority of the ALHIV in Ibadan have inadequate oral health knowledge and engage in poor oral health practices. On the other hand, a higher percentage of them had a good attitude towards oral health. The strength of this study lies in its providing baseline data on the oral health knowledge and attitudes of ALHIV, which have hitherto not been available. This data will help in developing comprehensive oral health promotional programmes that will foster oral health literacy and empower these adolescents to carry out appropriate preventive oral self-care habits in line with one of the key implementation strategies of the National Oral Health Policy (2024–2029) [15]. The findings of this study may apply to ALHIV receiving care in similar urban HIV care settings in low- and middle-income countries.

However, the study is not without its limitations. Being a questionnaire-based study with a cross-sectional design, it is subject to potential biases such as recall bias and social desirability bias. To assuage these concerns, participants were assured of anonymity and informed that their responses would not be traceable to them. In addition, since the aim of the study was not to establish an association between the HIV status of the participant and their OHKAP, no control group was included. We recommend that future studies may consider the incorporation of an appropriate comparative group to distinguish the effects of HIV status from those of age and other sociodemographic characteristics.

Six out of every ten participants had received prior information about oral health care, and the majority of them reported the school as the source of the information. This is understandable and expected, as oral health researchers have always advocated for oral health education in schools [22, 23].

Despite exposure to previous Oral Health Education (OHE) by a large proportion of the participants of this study, a very low mean oral health knowledge score was recorded, with very few scoring above half of the total obtainable score. Most of the adolescents could not correctly list the causes of dental caries and gingival (gum) diseases. In addition, the majority had not heard about fluoride, and only one participant knew the correct benefit or function of fluoride. Meanwhile, both dental caries and gingival/ periodontal disease have been reported to be common in this group of adolescents [3, 24]. This finding may be attributed to the nature of the oral health education (OHE) received, which may not have been sufficiently comprehensive and was possibly delivered as a one-off intervention without ongoing reinforcement. Evidence suggests that repeated and reinforced OHE is more effective in enhancing knowledge retention and promoting sustained improvements in oral health behaviours [25, 26]. It also suggests that school-based oral health education alone may not be sufficient to enlighten and empower this cohort of adolescents considering their increased risk of oral diseases. Furthermore, less than 10% of the participants had ever received oral health education from a dental or general hospital. This underscores an urgent need for educational intervention in this group, and paediatric infectious disease clinics can be a good site for this cohort as they visit at regular intervals for medical follow-up and medication collection.

Comparison of the findings on OHK and OHA and their associated factors in the present study with those of studies among adolescents with similar systemic conditions was not possible because of the scarcity of literature in this area. However, comparison of the OHK findings with those of school-based studies conducted in similar settings in Nigeria revealed similarly high levels of inadequate oral health knowledge [17, 22]. On the contrary, some other studies from another state in Nigeria and Africa at large revealed that a higher proportion of adolescents had higher oral health knowledge [23, 27–29]. This disparity in findings could be due to differences in school curricula between countries and states, which has been previously noted [28, 30]. 

The assessment of factors associated with adequacy of oral health knowledge among in-school adolescents has generated conflicting reports. A significant association between gender and level of knowledge favouring females was previously reported [17, 23, 27]. In this study, although the adjusted odds ratio suggested that males may have higher odds of adequate knowledge, the confidence interval was wide, and it included the null value, indicating substantial uncertainty around the estimate. Therefore, although this finding is similar to that of a previous study, it should be interpreted cautiously [31]. The differences in the oral health knowledge assessment tools used in these studies may also account for the disparity in findings.

Similarly, participants whose parents did not have tertiary education or were engaged in unskilled occupations had lower adjusted odds of having adequate oral health knowledge compared with their counterparts. This finding is consistent with previous reports [28]. This observation is understandable, as parental educational attainment, occupation, and socioeconomic status are known to influence family values, health literacy, and health-related behaviours [32, 33]. Although the direction of the associations was consistent with existing evidence, the present study did not provide strong evidence of an independent association between parental socioeconomic characteristics and oral health knowledge.

Compared with the OHK score, a higher mean OHA score was observed in the present study, with more than half of the participants demonstrating a positive attitude towards oral health. This finding is encouraging and suggests a potential opportunity for targeted oral health education, as a positive attitude may facilitate the adoption of appropriate oral health practices. Several factors could be responsible for this finding; one is the influence of social desirability bias, whereby participants may have provided responses they perceived to be socially acceptable or expected by healthcare providers. Additionally, regular interactions with healthcare professionals and repeated counselling sessions as part of HIV care may have fostered favourable attitudes towards oral health, even if these attitudes did not translate into adequate knowledge or sustained oral health behaviours. Cultural norms and beliefs may also shape attitudes differently from knowledge and practices, resulting in a disconnect between what participants perceive as desirable and their actual understanding and behaviours.

The mean OHA score in this present study is higher than that of previous school-based studies in the same environment [17, 22]. The better oral health attitude observed among adolescents living with HIV may also be attributable to their repeated exposure to counselling sessions, which form an integral component of the comprehensive care that the majority of the participants, having had vertically transmitted HIV infection, have received since childhood. Regular interactions with healthcare providers and continuous health education may have positively influenced their perceptions and attitudes towards oral health. Additionally, differences in the instruments and scoring methods used to assess oral health attitude across studies may partly explain the variations in findings.

In the final model, participants in the younger age group and those in the upper socioeconomic class were about 2 times as likely to have a positive attitude. In contrast, those whose parents had no tertiary education were less likely to possess a positive attitude compared to their counterparts. This still buttresses the influence of parental factors on a child’s attitude and beliefs. These influences are even better appreciated at a younger age before the child’s personal values are formed [32, 33]. 

Concerning oral health practices, less than half of the participants in this study had good oral health practices, which is similar to that of previous studies [7, 17, 22, 23, 34]. All the participants reported the use of a toothbrush, but only about 2 out of every ten participants used the appropriate quantity of toothpaste and brushed their teeth at the right time, after breakfast and dinner. Similarly, less than 10% of them used dental floss. This indicates an urgent need for oral health educational intervention in this population, considering that the use of the appropriate tool, frequency, and technique of toothbrushing is necessary for adequate plaque control [35, 36]. These findings are similar to previous reports [7, 9]. In addition, toothbrushes are not to be shared among family members, but in this present study, some participants still share their toothbrushes with their siblings. This needs to be highly discouraged, especially considering that toothbrushes have been shown to harbor micro-organisms such as *S. aureus* and Herpes Simplex viruses, which may be transferred to others [37–39].

Similar to the findings with OHK, males had higher odds of having good oral health practices and this could be because knowledge and practice have been shown to be related although, some studies have reported otherwise [40, 41]. Participants in the younger age group and those in primary schools had lesser odds of having good oral health practices, this may not be surprising as those in this category may not be old enough to carry out appropriate oral health practices if untrained and without appropriate guidance. This emphasizes the need to instil good oral health practices in children at a very young age. Similarly, lower parental educational attainment and engagement in unskilled occupations were associated with reduced odds of good oral health practices among participants. These findings may reflect the influence of parental socioeconomic circumstances on health-related behaviours and access to oral health information and resources. However, the estimates were imprecise, as evidenced by the wide confidence intervals that encompassed the null value. Consequently, this observed associations should be interpreted cautiously and cannot be considered conclusive evidence of an independent relationship after accounting for other factors.

## Conclusion and recommendation

In conclusion, this study found that although more than half of adolescents living with HIV in Ibadan, Nigeria, had participated in school-based oral health education, the majority of them still had inadequate knowledge of oral health and engaged in poor oral health practices. However, most of them had a good attitude towards oral health. Age was the only sociodemographic factor significantly associated with oral health attitude with younger children having better oral health attitude. Therefore, the findings highlight important gaps in oral health knowledge and practices among ALHIV and suggest the need for targeted oral health promotion and education amongst them.

## Data Availability

The datasets used and/or analyzed during the current study are available from the corresponding author on reasonable request.

## Abbreviations

ALHIV: Adolescents living with HIV infection
CALHIV: Children and Adolescents living with HIV infection
HAART: Highly Active Antiretroviral Therapy
HIV: Human Immunodeficiency Virus
OHA: Oral Health Attitude
OHE: Oral Health Education
OHK: Oral Health Knowledge
OHKAP: Oral Health Knowledge, Attitude and Practice
OHP: Oral Health Practices
SD: Standard deviation
